# Unravelling potential biomarkers for acute and chronic brucellosis through proteomic and bioinformatic approaches

**DOI:** 10.3389/fcimb.2023.1216176

**Published:** 2023-07-13

**Authors:** Yuejie Yang, Kunyan Qiao, Youren Yu, Yanmei Zong, Chang Liu, Ying Li

**Affiliations:** ^1^ Department of Infectious Diseases, Tianjin Second People’s Hospital, Tianjin, China; ^2^ Tianjin Institute of Hepatology, Tianjin Second People’s Hospital, Tianjin, China; ^3^ School of Medicine, Nankai University, Tianjin, China

**Keywords:** brucellosis, biomarkers, proteomics, bioinformatics, differential expression analysis, weighted gene co-expression network analysis (WGCNA), random forest model, enrichment analysis

## Abstract

**Introduction:**

This study aimed to identify biomarkers for acute and chronic brucellosis using advanced proteomic and bioinformatic methods.

**Methods:**

Blood samples from individuals with acute brucellosis, chronic brucellosis, and healthy controls were analyzed. Proteomic techniques and differential expression analysis were used to identify differentially expressed proteins. Co-expression modules associated with brucellosis traits were identified using weighted gene co-expression network analysis (WGCNA).

**Results:**

763 differentially expressed proteins were identified, and two co-expression modules were found to be significantly associated with brucellosis traits. 25 proteins were differentially expressed in all three comparisons, and 20 hub proteins were identified. Nine proteins were found to be both differentially expressed and hub proteins, indicating their potential significance. A random forest model based on these nine proteins showed good classification performance.

**Discussion:**

The identified proteins are involved in processes such as inflammation, coagulation, extracellular matrix regulation, and immune response. They provide insights into potential therapeutic targets and diagnostic biomarkers for brucellosis. This study improves our understanding of brucellosis at the molecular level and paves the way for further research in targeted therapies and diagnostics.

## 1 Introduction

Brucellosis, caused by gram-negative bacteria of the genus Brucella, is a zoonotic disease with a global impact (Shakir, 2021). Annually, it affects approximately half a million people, and it poses a significant public health challenge, particularly in developing countries where it is prevalent in both animals and humans (Hisham and Ashhab, 2018). The consumption of unpasteurized dairy products remains the primary mode of transmission (Rizkalla et al., 2021). Brucellosis is a significant public health issue in the Mediterranean region, the Middle East, Africa, Latin America, and parts of Asia (Mirnejad et al., 2017; Munyua et al., 2021). In China, the number of brucellosis patients has been increasing (Li et al., 2023). In 2021, there were 69,767 reported cases of Brucella infection with an incidence rate of 4.95 per 100,000, which is an increase of 22,522 cases compared to the previous year, representing a 47.7% increase (National Overview of Statutory Infectious Disease Epidemic Situation). This poses a significant threat to human health and overall socioeconomic development. Brucellosis can manifest as either acute or chronic forms, and both presentations share non-specific clinical symptoms that can mimic other infectious diseases, such as typhoid fever, rheumatic fever, osteoarthritis, and other diseases; hence it complicates accurate diagnosis and treatment (Zheng et al., 2018; Gentilini et al., 2019).

Given the heterogeneous and nonspecific clinical manifestations of brucellosis, laboratory confirmation is essential for accurate diagnosis (Yagupsky et al., 2019). Conventional diagnostic methods include culture, serology, and molecular techniques; however, these approaches exhibit limitations, such as suboptimal sensitivity, high costs, and lengthy turnaround times (Manzulli et al., 2022). Recently, proteomics and bioinformatics have emerged as potent tools for identifying novel biomarkers, providing potential advantages for the diagnosis, prognosis, and treatment of infectious diseases (Hamidi et al., 2022; Aggarwal et al., 2023; Mu et al., 2023). The analysis of circulating proteins in blood provides promising possibilities for diagnosis, risk stratification, and potentially prevention of diseases (Deutsch et al., 2021). Cristiana Iosef et al. suggested a vascular proliferative process in long COVID by analyzing the plasma proteome of long-COVID patients (Iosef et al., 2023). New proteomic methods, such as data independent acquisition mass spectrometry (DIA-MS), have recently surfaced as a valuable technique for the detection of blood-based biomarkers (Scott et al., 2023). In this study, we applied data dependent acquisition (DDA) and data independent acquisition mass (DIA) proteomic analysis to blood samples obtained from individuals with acute brucellosis, chronic brucellosis, and healthy controls. Our aim was to discover potential biomarkers that can effectively discriminate between acute and chronic brucellosis cases, as well as differentiate brucellosis patients from healthy individuals.

**Figure 1:**
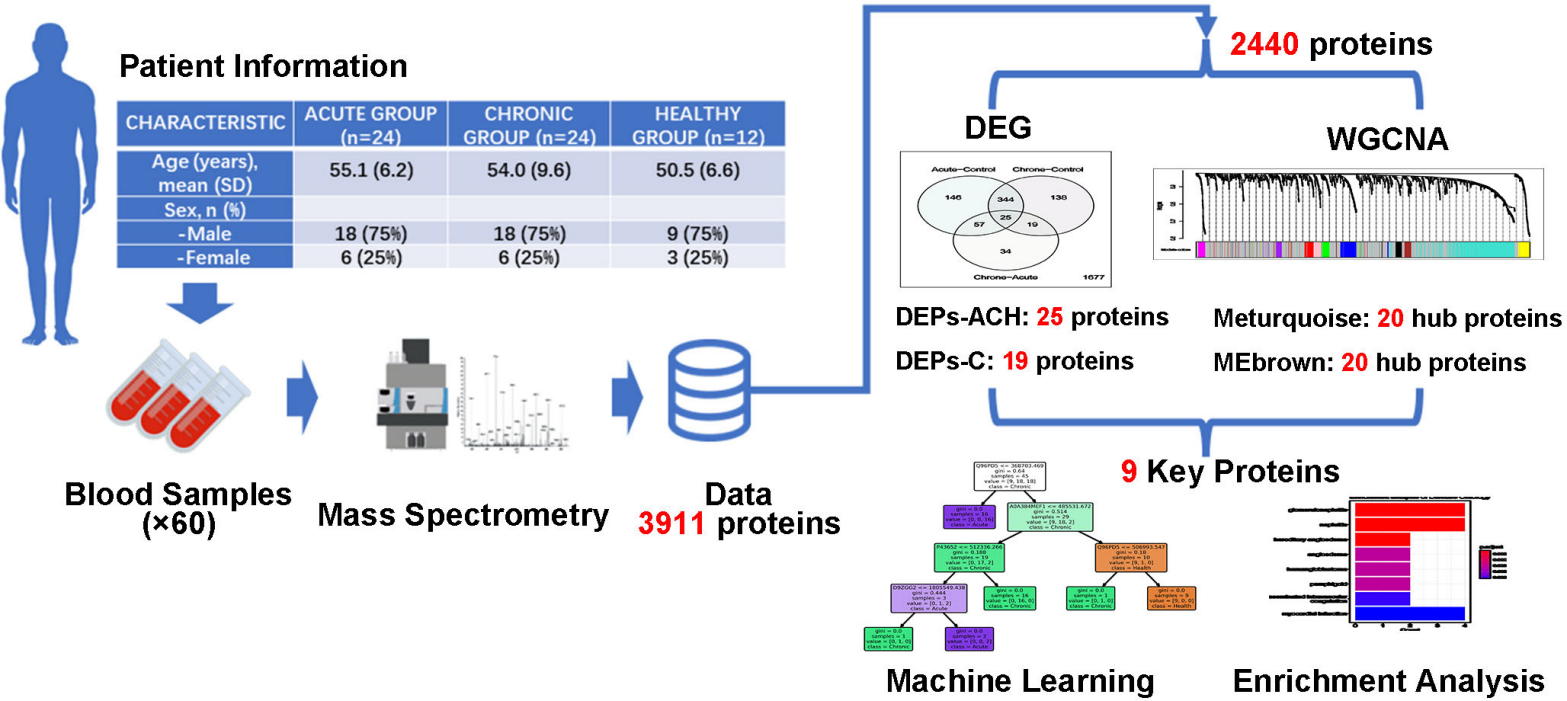
Flow chart of present study. Identification of key proteins in brucellosis. Serum samples were collected from 24 acute brucellosis patients, 24 chronic brucellosis patients, and 12 healthy controls. High-performance liquid chromatography-mass spectrometry identified 3,911 proteins, with 2,440 proteins expressed in all groups. Differential expression analysis and weighted gene co-expression network analysis (WGCNA) were performed, resulting in 9 key proteins being identified. Machine learning classification models and biological enrichment analysis were used to investigate the role of these proteins in brucellosis progression.

In this study as shown in **Figure 1**, a total of 60 participants were enrolled, comprising of 24 individuals with acute brucellosis, 24 individuals with chronic brucellosis, and 12 healthy controls. Serum samples were collected from each participant, and a high-performance liquid chromatography-mass spectrometry system was used to analyze the expression of proteins in the serum. A total of 3,911 proteins were identified, out of which 2,440 proteins were found to be expressed in all three groups. These 2,440 proteins underwent Differential Expression Analysis and Weighted Gene Co-Expression Network Analysis (WGCNA). The former identified two sets of important differentially expressed proteins, with 25 and 19 proteins, respectively, while the latter identified two modules that were significantly associated with clinical traits. Top 20 hub proteins were identified from each module, and nine key proteins were finally identified based on the results of the analyses. Machine learning classification models and biological enrichment analysis were then performed to further explore the potential significance of these proteins in the progression of brucellosis.

## 2 Materials and methods

### 2.1 Study populations

This research belongs to a cross-sectional study, and the study population consisted of 60 individuals from three groups: acute brucellosis (24 persons), chronic brucellosis (24 persons), and healthy controls (12 persons). The sample size was estimated using the formula $n={\left(\frac{Z_{\alpha /2}+Z_{\beta }}{\delta }\right)}^{2}\times p\times \left(1-p\right)$ . The male to female ratio in all groups was 3:1. All the participants were described in detail in **Supplementary Table 1**. The diagnosis of brucellosis was made according to the “Diagnostic criteria for brucellosis” (WS 269-2019) standard (Diagnosis for brucellosis), which included a history of brucellosis epidemiology, positive blood culture for Brucella, or positive Rose Bengal plate agglutination test with a tube agglutination test titer of 1:100 or higher. Acute brucellosis was defined as a history of illness within six months, while chronic brucellosis was defined as a history of illness for more than six months. Patients with other serious internal diseases or with other infectious diseases such as typhoid, paratyphoid, rheumatic fever, pulmonary tuberculosis, malaria, were excluded. Individuals considered inappropriate for inclusion in this study by the researchers were also excluded. The healthy control group population was composed of individuals who underwent health checkups.

### 2.2 Sample preparation and fractionation for data dependent Acquisition (DDA) library generation

According to the method of previous research (Wisniewski et al., 2009), we separated serum or plasma pools into high and low abundance protein fractions using a commercially available Human 14/Mouse 3 Multiple Affinity Removal System Column (Agilent Technologies), and concentrated the samples using a 5 kDa ultrafiltration tube (Sartorius). Sodium Dodecyl Sulfate Dithiothreitol (SDT) buffer (4% SDS, 100 mM DTT, 150 mM Tris- HCl pH 8.0) was added to the protein samples, which were then boiled and centrifuged. The supernatant was quantified using a Bicinchoninic Acid (BCA) Protein Assay Kit (Bio-Rad, USA) and stored at -80°C. This method enables the detection of lower abundance proteins that are potentially important in disease diagnosis and prognosis.

### 2.3 Filter-aided sample preparation (FASP Digestion) procedure

For protein digestion, we followed the modified FASP protocol described previously (Wisniewski, 2018). Briefly, 200 μg of high abundant and low abundant proteins were subjected to repeated ultrafiltration using Urea-Containing (UA) buffer (8 M Urea, 150 mM Tris-HCl pH 8.0) to remove the detergent, Dithiothreitol (DTT), and low-molecular-weight components. The reduced cysteine residues were blocked by adding 100 μl of 100 mM Iodoacetamide (IAA) in UA buffer, followed by incubation in darkness for 30 min. The samples were washed with UA buffer and 25 mM NH_4_HCO_3_ buffer, and then digested with 4 μg of trypsin in 40 μl of 25 mM NH_4_HCO_3_ buffer overnight at 37°C. The resulting peptides were collected as a filtrate and desalted on C18 cartridges, concentrated, and reconstituted in 0.1% formic acid. To fractionate the peptides, we used the High pH Reversed-Phase Peptide Fractionation Kit and collected 10 fractions. The peptides from the low-abundance components of serum/plasma samples were fractionated. Each fraction was concentrated, desalted on C18 cartridges, and reconstituted in 0.1% formic acid. To correct the relative retention time differences between runs, we added iRT-Kits (Biognosys) with a volume proportion of 1:3 for iRT standard peptides versus sample peptides.

### 2.4 Data dependent acquisition (DDA) mass spectrometry assay

For DDA library generation, all fractions were analyzed on a Thermo Scientific Q Exactive HF X mass spectrometer connected to an Easy nLC 1200 chromatography system (Thermo Scientific). Briefly, 1.5 μg of peptide was loaded onto an EASY-SprayTM C18 Trap column (Thermo Scientific) and separated on an EASY-SprayTM C18 LC Analytical Column (Thermo Scientific) with a linear gradient of buffer B (84% acetonitrile and 0.1% Formic acid) at a flow rate of 250 nl/min over 120 min. The Mass Spectrometry (MS) detection method was positive ion, and the scan range was 300-1800 m/z with a resolution of 60000 at 200 m/z for MS1 scan. The Automatic Gain Control (AGC) target was 3e6, and the maximum IT was 25ms, with dynamic exclusion set at 30.0s. Each full Mass Spectrometry Selected Ion Monitoring (MS-SIM) scan was followed by 20 ddMS2 scans, and the resolution for MS2 scan was 15000, with an AGC target of 5e4, maximum Injection Time (IT) of 25 ms, and normalized collision energy of 30 eV.

### 2.5 Mass spectrometry assay for data independent acquisition (DIA)

For liquid chromatography tandem mass spectrometry (LC-MS/MS) analysis in the data-independent acquisition (DIA) mode, peptides from each sample were analyzed by Shanghai Applied Protein Technology Co., Ltd. A DIA cycle included one full MS–SIM scan, with 30 DIA scans covering a mass range of 350–1800 m/z. SIM full scan resolution was set at 120,000 at 200 m/z with AGC at 3e6 and a maximum IT of 50ms. DIA scans were performed at a resolution of 15,000, with AGC target at 3e6 and maximum IT set to auto. Normalized collision energy was set at 30 eV. The runtime was 120 min with a linear gradient of buffer B (84% acetonitrile and 0.1% Formic acid) at a flow rate of 250 nl/min. To monitor MS performance, Quality Control (QC) samples were injected with DIA mode at the beginning of the MS study and after every 6 injections throughout the experiment.

### 2.6 Mass spectrometry data analysis

For the generation of DDA library data, the Uniprot human database was searched using Spectronaut ™ (14.4.200727.47784) software with the addition of indexed Retention Time (iRT) peptides sequence. The search parameters included trypsin as the enzyme, 2 as the maximum missed cleavages, carbamidomethyl(C) as the fixed modification, and oxidation(M) and acetyl (Protein N-term) as the dynamic modifications. The protein identification was determined by a false discovery rate (FDR) of ≤ 1% based on 99% confidence. The original raw files and DDA searching results were imported into Spectronaut Pulsar X™ (12.0.20491.4) for the construction of the spectral library. For the DIA data, the constructed spectral library was searched using Spectronaut™ (14.4.200727.47784) software with dynamic iRT as the retention time prediction type, enabled interference on MS2 level correction, and enabled cross-run normalization. The results were filtered based on a Q value cutoff of 0.01, equivalent to FDR< 1%. The mass spectrometry proteomics data have been deposited to the ProteomeXchange Consortium (http://proteomecentral.proteomexchange.org) via the iProX partner repository (Ma et al., 2019; Chen et al., 2022) with the dataset identifier PXD042212.

### 2.7 Data pre-processing and differential expression analysis

Data pre-processing was conducted using R software (version 4.2.2) with the tidyverse package (version 1.3.2). Firstly, proteins/peptides with missing expression values in more than 15 samples out of the 60 total were eliminated. The resulting data were log-transformed, and differential expression analysis was performed using the limma package (version 3.44.3) between the groups of interest (Ritchie et al., 2015). Volcano maps and boxplot graphics were created using the ggplot2 package (version 3.4.0), while heatmaps were generated using the pheatmap package (version 1.0.12).

### 2.8 Weighted gene correlation network analysis (WGCNA)

In this study, we employed Weighted Gene Correlation Network Analysis (WGCNA) to identify distinct protein/peptide modules among the all identified proteins (Langfelder and Horvath, 2008). To construct the weighted protein co-expression network, we used the WGCNA package (version 1.71) and set the network type as an unsigned network, with Pearson correlation as the correlation method. More details on the parameters and cut-offs used, including the power of 2 and module size cut-off of 30. To analyze the network, we calculated and visualized the module eigengene expression, adjacency matrix heatmap, module-trait relationship matrix, and gene significance versus module membership analysis, following the recommended guidelines of the WGCNA package. We exported the networks of the top 20 hub proteins/peptides to VisANT, an integrative framework for networks in systems biology, and visualized the network connections among the most connected proteins/peptides in the significant modules using the VisANT software (version 5.53) (Granger et al., 2016).

### 2.9 Random forests learning

Random forest is a machine learning algorithm that combines the results of multiple decision trees to make predictions. In this study, we employed a random forest model to classify blood samples from acute brucellosis patients, chronic brucellosis patients and healthy individuals based on the different expression of the key 9 proteins/peptides. We trained the model with the scikit-learn (version 1.0.2) module on the Python (version 3.7.7) Anaconda (version 23.1.0) platform. The max depth of decision trees was set to 4. We randomly split the data into training and testing sets at a ratio of 3:1 to avoid overfitting. We measured the impact of each feature (key protein/peptide) on the accuracy of the model using the Mean Decrease Accuracy (MDA) method. Additionally, we evaluated the performance of the model using the area under the receiver operating characteristic (ROC) curve, which measures the trade-off between the true-positive rate and the false-positive rate.

### 2.10 Analysis and visualization of functional enrichment

To investigate the biological functions and pathways associated with the key genes identified in our study, we performed an enrichment analysis using the clusterProfiler package (version 4.6.1) in R (Wu et al., 2021). We analyzed the gene ontology (GO) terms in three categories, including cellular component (CC), biological process (BP), and molecular function (MF), as well as Reactome pathways and disease ontology (DO) terms. We used the *enrichGO* function for GO analysis, *enrichPathway* function for Reactome pathway analysis, *enrichDO* function for DO analysis, and *barplot* function to visualize the results.

## 3 Results

### 3.1 Identification of differentially expressed proteins/peptides in acute and chronic brucellosis

**Figure 2:**
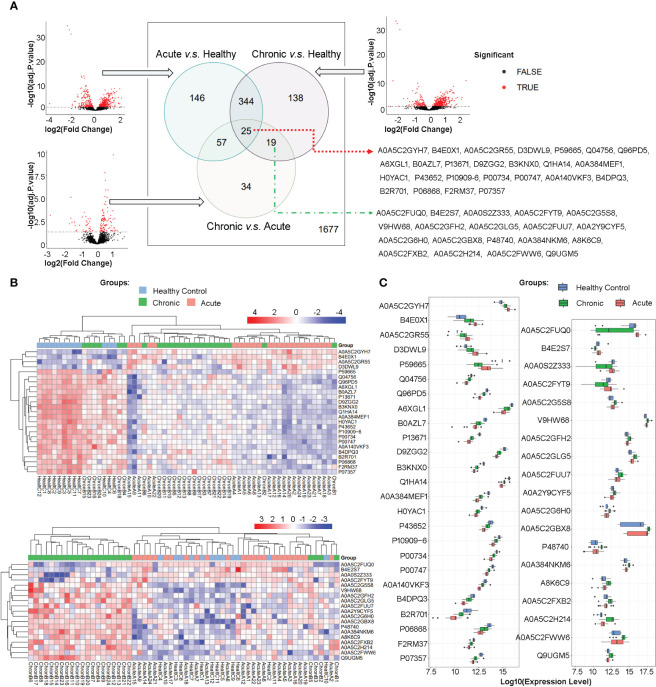
Differential Protein Expression in Plasma Proteome of Acute and Chronic Brucellosis Patients and Healthy Controls. **(A)** Volcano plots and Venn diagrams depicting differential protein expression in pairwise comparisons between the three groups. A total of 25 proteins/peptides were found to be significantly differentially expressed in all three pairwise comparisons. Moreover, 19 proteins/peptides were differentially expressed only in the comparisons between the chronic brucellosis group versus the healthy control group and the chronic brucellosis group versus the acute brucellosis group. The Uniprot accessions of these 25 and 19 proteins/peptides are provided. **(B)** Heatmaps demonstrating the expression patterns of the 25 and 19 differentially expressed proteins/peptides in plasma samples of the acute brucellosis group, the chronic brucellosis group, and the healthy control group. **(C)** Boxplots presenting the expression levels of the 25 and 19 differentially expressed proteins/peptides in plasma samples of the acute brucellosis group, the chronic brucellosis group, and the healthy control group.

High-throughput mass spectrometry data commonly contain missing values, and in this study, those proteins/peptides with missing expression values in more than 15 samples in all 60 samples were removed, resulting in 2440 proteins/peptides being analyzed. Differential expression analysis was performed using limma on the R platform, and volcano plots were used to visualize the significant differentially expressed proteins/peptides in three comparisons between the acute brucellosis group, the chronic brucellosis group, and the healthy control group. The overlap of differentially expressed proteins/peptides in the three comparisons was determined using Venn diagrams. As shown in **Figure 2A**, among the total 2440 proteins, 763 proteins were differentially expressed with statistical significance in pairwise comparisons between the three groups. Specifically, 146 proteins were differentially expressed between acute group and control group, 138 between chronic group and control group, and 34 between chronic group and acute group; 344 proteins were differentially expressed between acute vs control and chronic vs control group, 57 proteins were differentially expressed between acute vs control and chronic vs acute group, 19 proteins were differentially expressed between chronic vs control and chronic vs acute group; 25 proteins were differentially expressed in all three pairwise comparisons. We are particularly interested in the 25 proteins/peptides identified as differentially expressed in all three comparisons (abbreviated as DEPs-ACH). Additionally, 19 proteins/peptides were significantly differentially expressed only in the comparisons of the chronic brucellosis group versus the healthy control group and the chronic brucellosis group versus the acute brucellosis group (abbreviated as DEPs-C), which were considered as potential biomarkers for chronic brucellosis. The Uniprot accessions of DEPs-ACH and DEPs-C were listed in **Figure 2A**.

### 3.2 Expression patterns of DEPs-ACH and DEPs-C revealed by hierarchical clustering, heatmaps, and boxplots

In order to provide a detailed depiction of the expression patterns of the differentially expressed proteins/peptides (DEPs), we generated heatmaps and boxplots for DEPs-ACH and DEPs-C, as shown in **Figures 2B, C**. Hierarchical clustering of both samples and proteins was performed in the heatmaps, which revealed that in DEPs-ACH, samples could be divided into two main clusters, namely the healthy control cluster and the brucellosis cluster, with acute and chronic brucellosis samples well-dispersed within the brucellosis cluster. In contrast, the samples in DEPs-C clustered into a chronic brucellosis-specific cluster and another cluster comprising the healthy control and acute brucellosis samples. In the heatmaps of DEPs-ACH and DEPs-C, the DEPs were mainly clustered into two categories. In DEPs-ACH, the 4 proteins A0A5C2GYH7, B4E0X1, A0A5C2GR55, and D3DWL9 were highly expressed in the acute and chronic groups but lowly expressed in the control group, while the other 21 proteins showed the opposite trend. In DEPs-C, the 4 proteins A0A5C2FUQ0, B4E2S7, A0A0S2Z333, and A0A5C2FYT9 were highly expressed in the acute and control groups but lowly expressed in the chronic group, while the other 15 proteins showed the opposite trend. The expression changes in DEPs-ACH and DEPs-C were visually represented by the heatmaps, where the expression level of DEPs-ACH increased or decreased successively in the order of the healthy control group, chronic brucellosis group, and acute brucellosis group. In contrast, the expression of DEPs-C did not exhibit the same trend as DEPs-ACH, with only significant differential expression observed between the chronic brucellosis and the other two groups. To provide an overview of the variation in DEPs levels, boxplots were generated for DEPs-ACH and DEPs-C as shown in **Figure 2C**. Consistent with the heatmaps, the boxplots of DEPs-ACH showed a trend of increasing or decreasing expression levels from the healthy control group to the chronic brucellosis group and the acute brucellosis group, but P59665 was the only exception. Among the 25 DEPs-ACH, four DEPs (A0A5C2GYH7, B4E0X1, A0A5C2GR55, D3DWL9) exhibited increasing expression levels in the same order, while P59665 had the highest expression level in the healthy control group, the lowest in the chronic brucellosis group, and an intermediate level in the acute brucellosis group. The other 20 DEPs-ACH exhibited decreasing expression levels in the same order. Among the 19 DEPs-C, four DEPs (A0A5C2FUQ0, B4E2S7, A0A0S2Z333, A0A5C2FYT9) showed lower expression levels in the chronic brucellosis group than in the other two groups, while the other 15 DEPs-C exhibited higher expression levels in the chronic brucellosis group.

### 3.3 Identification of co-expression protein/peptide modules using WGCNA and their correlation with clinical traits in brucellosis patients

**Figure 3:**
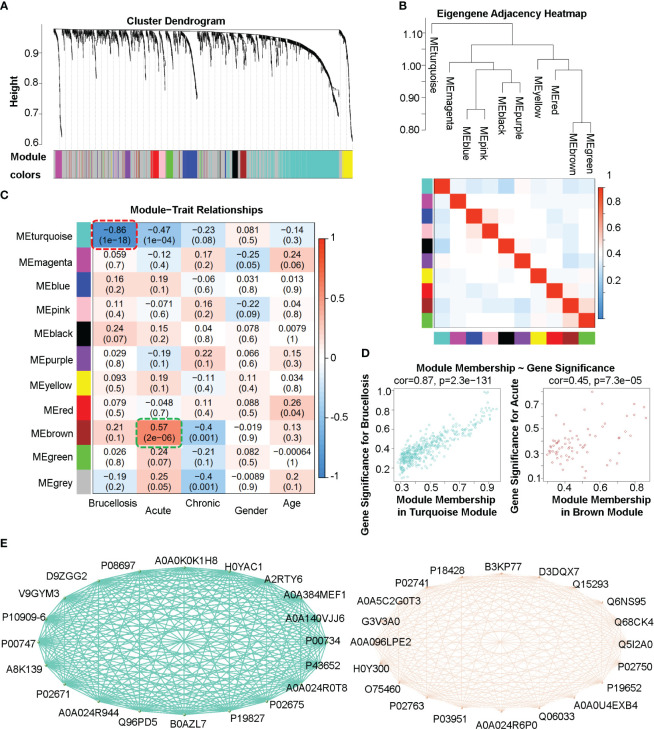
Weighted gene co-expression analysis (WGCNA) of the plasma proteome in patients with acute and chronic brucellosis and healthy controls. **(A)** Hierarchical clustering and module detection based on protein/peptide expression patterns. The dendrogram shows the clustering of proteins/peptides, and the colors below represent the 11 identified modules. **(B)** Dendrogram of consensus module eigengenes and heatmap of module adjacencies. The heatmap shows the correlations (positive or negative) between the identified modules. **(C)** Module-trait relationships for various clinical traits. The module name is shown on the left side of each cell, and the correlations between the module eigengene and each trait are displayed. The color-coded table indicates the strength of the correlations. **(D)** Scatter plots showing the relationship between gene significance and module membership in the turquoise (left) and brown (right) modules. **(E)** Network diagrams constructed using the top 20 hub proteins/peptides in the turquoise (left) and brown (right) modules. The Uniprot accessions and relationships between hub proteins/peptides are shown.

To identify co-expression protein/peptide modules, we utilized the WGCNA method to construct a weighted co-expression network from the 2440 filtered proteins/peptides. Eleven modules with different colors, including 10 co-expression modules and the grey module (MEgrey) for proteins/peptides outside all modules, were identified, as shown in **Figures 3A, B**. In **Figure 3C**, the module-trait relationship heatmap demonstrates the correlations between the module eigengene and clinical traits, while a module-trait relationship heatmap containing more clinical indexes were presented in the **Supplemental Figure 1**. Since we constructed an unsigned WGCNA network, the expression of proteins/peptides in the significant module changes significantly with the specific clinical trait. The results indicate that the turquoise module is negatively correlated with brucellosis infection and clinical symptoms such as fatigue, fever, muscle aches, and low back pain; while there is a positive correlation between the brown module and acute brucellosis infection, as indicated by the red dashed box and green dashed box in **Figure 3C**. Moreover, we analyzed the gene significance for brucellosis of the proteins/peptides in the turquoise module and the gene significance for acute infection of the proteins/peptides in the brown module, as presented in **Figure 3D**. The higher module membership proteins/peptides in the module are more significant for the corresponding clinical trait, so the hub proteins/peptides that are highly connected in the module are considered as hub genes according to the naming convention of WGCNA. We exported the networks consisting of the top 20 hub genes (proteins/peptides actually) of the turquoise and brown modules to the VisANT software, and visualized the Uniprot accessions and network relations of the top 20 hub proteins/peptides of the turquoise and brown modules in **Figure 3E**. These hub genes are critical components of network structures and functions, and usually have significant biological functions and association with traits of interest.

### 3.4 Identification and classification of key proteins associated with brucellosis and enrichment analysis of biological functions

**Figure 4:**
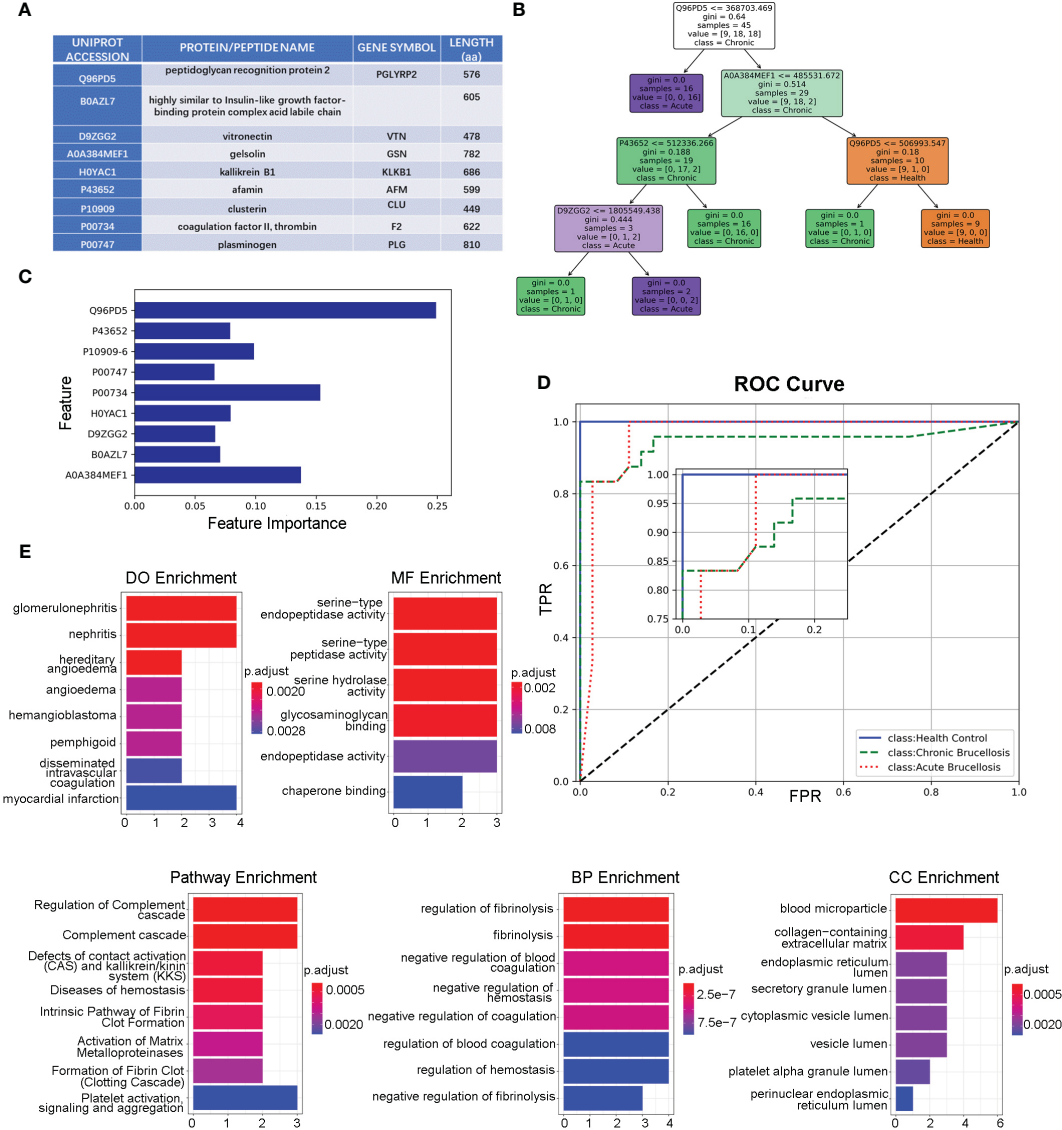
Functional Analysis of the key nine proteins/peptides. **(A)** Summary of key 9 proteins/peptides: This table provides detailed information on the 9 proteins/peptides identified as potential biomarkers for brucellosis in our study, including Uniprot accession, protein/peptide name, gene symbol, and amino acid sequence length. **(B)** Decision Tree Example in Random Forest Model: This figure illustrates an example of a decision-making tree generated by our random forest model for predicting brucellosis. The tree is based on the most important features identified in our analysis and shows the decision rules for classifying samples as acute brucellosis, chronic brucellosis, or healthy. **(C)** Feature Importance in Forest Model: This figure shows the feature importance scores for the 9 key proteins/peptides in our random forest model. The x-axis represents the importance scores, and the y-axis shows the proteins. **(D)** ROC Curves of Forest Model: This figure presents the ROC curves of the three different classes for our random forest model. The x-axis displays the false-positive rate, and the y-axis shows the true-positive rate. The area under the curve (AUC) values for all three classes are above 0.95, indicating that our model has good discriminatory power. **(E)** Enrichment Analysis of the Key 8 Genes: This section displays the results of the enrichment analysis of the 8 key genes, including DO analysis, MF analysis, pathway analysis, BP analysis, and CC analysis.

We identified key proteins by intersecting differentially expressed proteins (DEPs-ACH and DEPs-C) with hub proteins (MEturquosis and MEbrown) from WGCNA. This analysis eventually yielded 9 key proteins that overlapped with DEPs-ACH and the hub proteins of MEturquosis. **Figure 4A** displays the relevant information for these 9 proteins, with all except for B0AZL7 having corresponding gene symbols, while a more detailed information table was presented in the **Supplemental Table 2**. Furthermore, these nine proteins were used to train a random forest model for classifying samples into three categories: acute brucellosis, chronic brucellosis, and healthy control. **Figures 4B, C** show one decision tree of the model and feature importance, respectively. As shown in the feature importance chart, Q96PD5 was the most important feature in classification. Q96PD5(PGLRP2) is a peptidoglycan recognition protein, which belongs to the N-acetylmuramoyl-L-alanine amidase 2 family. As shown in **Figure 4D**, ROC curves for the three different categories, with all three having an AUC greater than 0.95, indicating good classification performance. By zooming in on the plot, we can clearly see that the healthy control group is better than the acute brucellosis group, which in turn is better than the chronic brucellosis group. To investigate the biological functions of the nine key proteins identified in the previous analysis, we performed enrichment analysis using the clusterProfiler package in R. Since B0AZL7 lacks a corresponding gene name, we analyzed eight genes in total. Enrichment analysis was performed for gene ontology (GO) terms in three categories, including cellular component (CC), biological process (BP), and molecular function (MF), as well as Reactome pathways and disease ontology (DO) terms. The results of the analysis are presented in **Figure 4E**. The enrichment analysis revealed that these proteins are primarily concentrated in blood microparticles, which is consistent with the serum samples used in the study. Additionally, the proteins were found to have molecular functions mainly involving serine-type peptidase activity and were implicated in regulating fibrinolysis and complement cascade processes. The proteins were also associated with nephritis, as indicated by the DO term analysis.

## 4 Discussion

In the present investigation, we employed advanced proteomic and bioinformatic methodologies to scrutinize blood specimens derived from individuals with acute and chronic brucellosis, alongside healthy controls, with the aim of uncovering potential biomarkers associated with brucellosis. Our findings unveiled a distinct set of differentially expressed proteins, which hold promise as diagnostic or prognostic indicators for brucellosis. Furthermore, these molecular signatures may provide valuable insights into the underlying mechanisms and pathological processes involved in brucellosis pathogenesis.

The endeavor of identifying pivotal proteins is intrinsically enlightening. In this investigation, we utilized two prevalent approaches in the realm of biological informatics: differential expression analysis and weighted gene co-expression network analysis (WGCNA). Through differential expression analysis, we discerned 25 proteins within the DEPs-ACH dataset, exhibiting significant differential expression across all three pairwise comparisons among the three groups. Concurrently, WGCNA analysis revealed 20 hub proteins in the MEturquoise module, demonstrating a strong association with the clinical manifestations of brucellosis.

Proteomic studies related to brucellosis reported to date have primarily focused on the investigation of Brucella-specific antigens and their interaction with host antibodies. Gamal Wareth et al. performed a comprehensive identification of immunodominant proteins in *Brucella abortus* and *Brucella melitensis* using antibodies present in sera from naturally infected hosts (Wareth et al., 2016). Ayman Elbehiry et al. conducted proteomics-based screening and antibiotic resistance assessment of clinical and sub-clinical Brucella species (Elbehiry et al., 2022). Meijuan Pei et al. utilized mass spectrometry in conjunction with the collection of major histocompatibility complex class I or II (MHC-I/II)-binding peptides from blood samples for the identification of potential antigenic peptides of Brucella (Pei et al., 2023). In our study, we aimed to compare patients with acute and chronic brucellosis infections to a healthy control group, with the objective of identifying distinctive key proteins or peptides in the blood. Ideally, these proteins would possess discriminatory characteristics capable of distinguishing between acute and chronic infections.

Based on the aforementioned aim, employing two bioinformatics analyses, we ultimately obtained nine proteins, thereby offering mutual validation of the reliability of these techniques, and underscoring the crucial role of these proteins in brucellosis progression. The overlap indicates that these shared key proteins likely serve as central players in the biological processes, with their regulation potentially impacting brucellosis. It is imperative to acknowledge the complementary nature of WGCNA and differential expression analysis. WGCNA is geared towards the identification of gene clusters with analogous expression patterns, subsequently unveiling potential functional modules and gene interactions (Kakati et al., 2019). In contrast, differential expression analysis is primarily concerned with significantly differentially expressed genes under diverse conditions to pinpoint key genes linked to specific biological processes or states (Deshpande et al., 2023). Hence, the integration of these two analytical methodologies can yield a more comprehensive understanding of the regulatory networks and molecular mechanisms governing biological processes.

Drawing upon the expression levels of the nine proteins previously mentioned, we constructed a multi-class random forest model. This machine learning approach, predicated on the integration of numerous decision trees, circumvents challenges associated with overfitting and inadequate generalization capabilities. The model can be applied directly to data, boasts commendable interpretability, and can rank the significance of the provided features, among other merits. The ROC curve analysis substantiates the model’s efficacy in accurately classifying the healthy group, acute brucellosis group, and chronic brucellosis group. Feature importance analysis reveals that the top three salient features, in descending order, comprise Q96PD5 (PGLYRP2), P00734 (F2), and A0A384MEF1 (GSN). Q96PD5, also referred to as peptidoglycan recognition protein 2, is a member of a protein class, peptidoglycan recognition proteins (PGRP), which possess the ability to bind or hydrolyze peptidoglycan (PGN). PGRP-S-PGN complexes augment the membrane expression of CD14, CD80, and CD86 while amplifying the secretion of interleukin-8, interleukin-12, and tumor necrosis factor-alpha. Conversely, these complexes diminish interleukin-10, thereby manifesting a distinct inflammatory profile (De Marzi et al., 2015). P00734 (F2) is proteolytically cleaved in multiple steps to form the activated serine protease thrombin, and thrombin also plays a role in cell proliferation, tissue repair, and angiogenesis. A0A384MEF1 (GSN) is a calcium-regulated protein functions in both assembly and disassembly of actin filaments. Based on the functional characteristics of these proteins, we speculate that these proteins may be involved in the immune response of the host and cellular damage processes during brucellosis infection.

Among the nine proteins previously mentioned, eight possess corresponding gene designations. We conducted an enrichment analysis on the following genes: PGLYRP2, VTN, GSN, KLKB1, AFM, CLU, F2, and PLG. This comprehensive enrichment analysis encompasses Gene Ontology (GO) assessments, which examine Cellular Component (CC), Biological Process (BP), and Molecular Function (MF) categories, as well as Reactome Pathway analysis and Disease Ontology (DO) investigation. The enrichment analysis results elucidate that the investigated genes predominantly participate in an array of biological processes, encompassing inflammation, coagulation, and extracellular matrix regulation. The implication in glomerulonephritis and nephritis alludes to a potential function in renal-associated inflammatory conditions. Currently, there have been reports linking glomerulonephritis/nephritis with brucellosis. One such report describes immune complex-mediated glomerulonephritis occurring in a patient (29-year-old man) with concurrent brucellosis following COVID-19 vaccination with the AstraZeneca vaccine (Al Bakr and Alaithan, 2022). Additionally, there have been several reports documenting cases of glomerulonephritis attributed to brucellosis (Parlak, 2020; Shebli et al., 2021). Moreover, the enrichment in processes such as hereditary angioedema, complement cascade, and platelet activation signaling insinuates a nexus with immune response and blood coagulation regulation. The correlation with extracellular matrix constituents, including glycosaminoglycan binding and collagen-rich extracellular matrix, underscores their potential role in tissue remodeling and cellular adhesion. In summary, the multifaceted enrichment of these genes indicates a possible contribution to an extensive spectrum of physiological and brucellosis pathological processes, thereby offering invaluable insights into potential therapeutic targets or diagnostic biomarkers for brucellosis.

Our study presents certain limitations, including a relatively small sample size and the absence of validation in a larger patient cohort. Based on the sample size formula and parameters, we obtained a sample size of 18 for each group. Due to limitations in the actual situation, our actual sample size for the healthy control group is 12, which is lower than the calculated required sample size of 18. However, the sample sizes for the acute group and chronic group are 24. The deficiencies may affect the results of the experiment, and we did not find the specific biomarkers that can effectively discriminate between acute and chronic brucellosis cases. Additionally, the analysis was restricted to blood samples, while other body fluids such as cerebrospinal or synovial fluid may harbor more specific biomarkers for brucellosis. Despite these limitations, our research highlights the potential of proteomics technology in discovering novel biomarkers for infectious diseases like brucellosis, which could ultimately enhance diagnosis, prognosis, and treatment. The random forest model based on nine key proteins demonstrated good classification performance for acute, chronic and health groups. Future investigations should focus on validating these biomarkers in larger patient cohorts and diverse body fluids, as well as conducting longitudinal studies to examine biomarker expression changes throughout the disease progression and treatment response. Ultimately, the identification of reliable and specific biomarkers for brucellosis is a crucial step towards developing efficacious diagnostic and therapeutic strategies for this overlooked disease.

## Data availability statement

The datasets presented in this study can be found in online repositories. The names of the repository/repositories and accession number(s) can be found below: The data presented in the study are deposited in the ProteomeXchange Consortium (http://proteomecentral.proteomexchange.org) repository, accession number PXD042212.

## Ethics statement

The studies involving human participants were reviewed and approved by Ethics Committee for Human Research of Tianjin Second People’s Hospital. The patients/participants provided their written informed consent to participate in this study.

## Author contributions

YJY, KQ, CL and YL contributed to conception and design of the study. YJY, KQ and YZ collected samples and organized the database. YRY and CL performed the statistical and bioinformatical analysis. YJY and CL wrote the first draft of the manuscript. KQ, YJY, YZ, and YL wrote sections of the manuscript. All authors contributed to the article and approved the submitted version.
