# Supplementary material for: Unravelling potential biomarkers for acute and chronic brucellosis through proteomic and bioinformatic approaches
Source: Front Cell Infect Microbiol. 2023 Jul 13;13:1216176. doi: 10.3389/fcimb.2023.1216176 (PMC10373591; doi:10.3389/fcimb.2023.1216176)
Supplement: Supplementary file 1 [file DataSheet_1.docx]

Supplementary Material

Unraveling Potential Biomarkers for Acute and Chronic Brucellosis Through Proteomic and Bioinformatic Approaches

Yuejie Yang^1#^, Kunyan Qiao^2#^, Youren Yu^3^, Yanmei Zong^1^, Chang Liu^3*^, Ying Li^1*^

*** Correspondence:** Chang Liu: [changliu@nankai.edu.cn](mailto:changliu@nankai.edu.cn); Ying Li: [liying9886@126.com](mailto:liying9886@126.com)

# Supplementary Figures

**
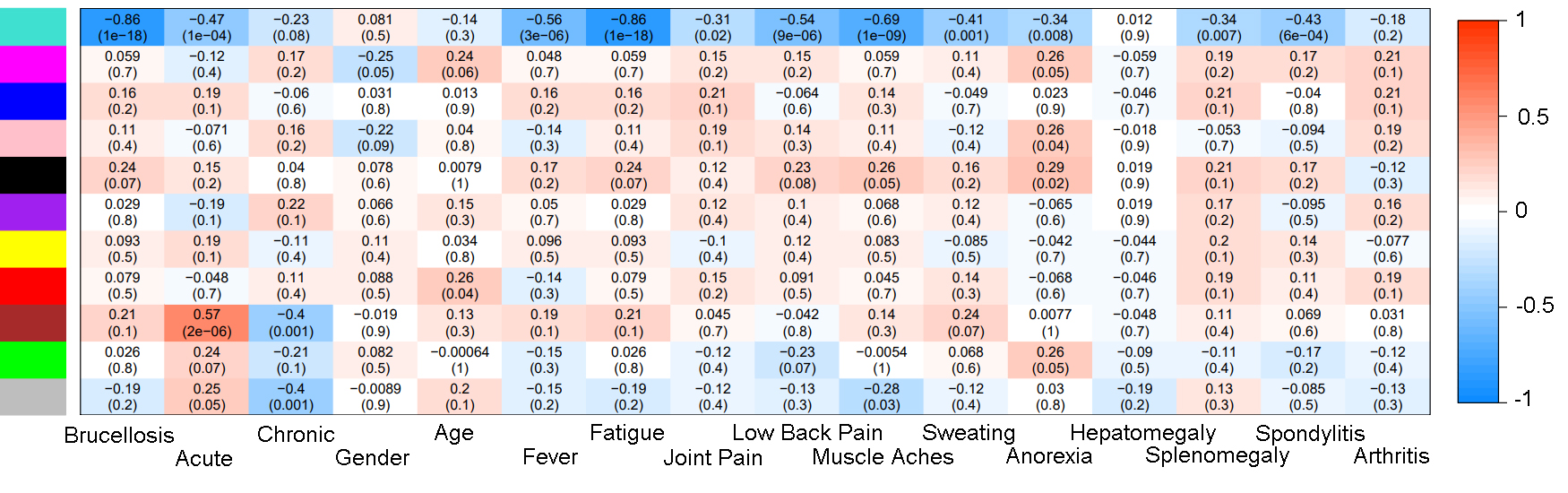
**

**Supplementary Figure 1.** Module-trait relationships for various clinical traits (Detailed). The module name is shown on the left side of each cell, and the correlations between the module eigengene and each trait are displayed. The color-coded table indicates the strength of the correlations.

# Supplementary Tables

**Supplementary Table 1. Patient Information**

| **Patient ID** | **Gender** | **Age** | **Fever** | **Fatigue** | **Joint Pain** | **Low Back Pain** | **Muscle Aches** | **Sweating** | **Anorexia** | **Hepatomegaly** | **Splenomegaly** | **Spondylitis** | **Arthritis** |
| --- | --- | --- | --- | --- | --- | --- | --- | --- | --- | --- | --- | --- | --- |
| **AcuteA1** | Male | 51 | 1 | 1 | 0 | 0 | 1 | 0 | 0 | 0 | 0 | 0 | 0 |
| **AcuteA2** | Female | 58 | 1 | 1 | 1 | 0 | 1 | 1 | 0 | 0 | 0 | 0 | 0 |
| **AcuteA3** | Female | 62 | 1 | 1 | 1 | 1 | 0 | 0 | 0 | 0 | 0 | 1 | 1 |
| **AcuteA4** | Female | 61 | 1 | 1 | 0 | 1 | 1 | 1 | 0 | 0 | 1 | 0 | 0 |
| **AcuteA5** | Male | 51 | 1 | 1 | 1 | 0 | 1 | 0 | 0 | 0 | 0 | 0 | 0 |
| **AcuteA6** | Female | 67 | 1 | 1 | 1 | 0 | 0 | 1 | 1 | 0 | 1 | 0 | 1 |
| **AcuteA7** | Male | 52 | 0 | 1 | 0 | 1 | 1 | 0 | 0 | 0 | 1 | 0 | 0 |
| **AcuteA8** | Female | 60 | 1 | 1 | 1 | 1 | 1 | 1 | 0 | 0 | 0 | 1 | 0 |
| **AcuteA9** | Male | 47 | 1 | 1 | 1 | 1 | 0 | 1 | 0 | 0 | 1 | 1 | 1 |
| **AcuteA10** | Male | 52 | 1 | 1 | 1 | 0 | 1 | 1 | 0 | 0 | 0 | 0 | 1 |
| **AcuteA11** | Male | 48 | 1 | 1 | 0 | 0 | 1 | 0 | 0 | 0 | 1 | 0 | 0 |
| **AcuteA12** | Male | 55 | 1 | 1 | 1 | 0 | 1 | 0 | 0 | 0 | 1 | 0 | 1 |
| **AcuteA13** | Male | 55 | 1 | 1 | 0 | 1 | 1 | 1 | 0 | 0 | 1 | 1 | 0 |
| **AcuteA14** | Male | 56 | 0 | 1 | 0 | 0 | 1 | 1 | 1 | 0 | 0 | 0 | 0 |
| **AcuteA15** | Male | 64 | 1 | 1 | 0 | 0 | 1 | 0 | 0 | 0 | 1 | 0 | 0 |
| **AcuteA16** | Male | 61 | 1 | 1 | 0 | 0 | 1 | 0 | 0 | 0 | 0 | 0 | 0 |
| **AcuteA17** | Male | 41 | 1 | 1 | 0 | 0 | 1 | 0 | 0 | 0 | 0 | 0 | 0 |
| **AcuteA18** | Female | 62 | 1 | 1 | 0 | 1 | 1 | 1 | 1 | 0 | 0 | 1 | 0 |
| **AcuteA19** | Male | 56 | 1 | 1 | 0 | 1 | 1 | 0 | 0 | 0 | 1 | 1 | 0 |
| **AcuteA20** | Male | 58 | 1 | 1 | 1 | 1 | 1 | 1 | 1 | 0 | 1 | 1 | 0 |
| **AcuteA21** | Male | 51 | 1 | 1 | 0 | 1 | 1 | 1 | 0 | 0 | 0 | 1 | 0 |
| **AcuteA22** | Male | 53 | 0 | 1 | 0 | 1 | 1 | 0 | 0 | 1 | 1 | 1 | 0 |
| **AcuteA23** | Male | 47 | 1 | 1 | 0 | 1 | 1 | 1 | 1 | 0 | 1 | 0 | 0 |
| **AcuteA24** | Female | 54 | 1 | 1 | 1 | 1 | 1 | 1 | 1 | 0 | 1 | 0 | 1 |
| **ChronB1** | Male | 48 | 1 | 1 | 1 | 1 | 1 | 1 | 0 | 0 | 0 | 1 | 1 |
| **ChronB2** | Female | 46 | 0 | 1 | 1 | 1 | 1 | 0 | 0 | 0 | 0 | 0 | 0 |
| **ChronB3** | Male | 45 | 1 | 1 | 0 | 1 | 1 | 1 | 0 | 0 | 0 | 1 | 0 |
| **ChronB4** | Male | 57 | 1 | 1 | 1 | 0 | 1 | 1 | 0 | 1 | 1 | 0 | 1 |
| **ChronB5** | Male | 55 | 1 | 1 | 1 | 1 | 1 | 0 | 0 | 0 | 0 | 1 | 1 |
| **ChronB6** | Male | 65 | 0 | 1 | 1 | 1 | 1 | 1 | 0 | 0 | 1 | 1 | 1 |
| **ChronB7** | Female | 53 | 0 | 1 | 1 | 1 | 1 | 0 | 1 | 0 | 0 | 0 | 1 |
| **ChronB8** | Male | 77 | 0 | 1 | 0 | 1 | 1 | 0 | 0 | 0 | 0 | 1 | 0 |
| **ChronB9** | Male | 48 | 1 | 1 | 1 | 1 | 1 | 1 | 0 | 0 | 1 | 1 | 0 |
| **ChronB10** | Female | 45 | 1 | 1 | 0 | 0 | 1 | 0 | 1 | 0 | 0 | 0 | 0 |
| **ChronB11** | Female | 67 | 1 | 1 | 1 | 1 | 1 | 1 | 1 | 0 | 1 | 1 | 1 |
| **ChronB12** | Male | 48 | 1 | 1 | 1 | 1 | 1 | 1 | 0 | 0 | 0 | 0 | 1 |
| **ChronB13** | Male | 52 | 1 | 1 | 1 | 1 | 1 | 1 | 1 | 0 | 0 | 1 | 1 |
| **ChronB14** | Male | 55 | 1 | 1 | 0 | 1 | 1 | 1 | 0 | 0 | 1 | 1 | 0 |
| **ChronB15** | Male | 36 | 1 | 1 | 1 | 1 | 1 | 1 | 0 | 0 | 0 | 1 | 1 |
| **ChronB16** | Male | 36 | 1 | 1 | 0 | 1 | 1 | 1 | 0 | 0 | 1 | 1 | 0 |
| **ChronB17** | Male | 58 | 0 | 1 | 0 | 1 | 1 | 0 | 0 | 0 | 0 | 0 | 0 |
| **ChronB18** | Male | 56 | 1 | 1 | 1 | 1 | 1 | 1 | 0 | 0 | 0 | 1 | 1 |
| **ChronB19** | Male | 62 | 0 | 1 | 1 | 1 | 1 | 0 | 0 | 0 | 1 | 0 | 1 |
| **ChronB20** | Female | 52 | 1 | 1 | 0 | 1 | 1 | 1 | 0 | 0 | 0 | 1 | 0 |
| **ChronB21** | Female | 64 | 1 | 1 | 1 | 1 | 1 | 1 | 1 | 0 | 0 | 1 | 0 |
| **ChronB22** | Male | 50 | 0 | 1 | 1 | 1 | 1 | 0 | 0 | 0 | 0 | 1 | 1 |
| **ChronB23** | Male | 56 | 1 | 1 | 1 | 1 | 1 | 0 | 0 | 0 | 0 | 0 | 1 |
| **ChronB24** | Male | 64 | 1 | 1 | 1 | 1 | 1 | 1 | 0 | 0 | 1 | 0 | 1 |
| **HealtC1** | Male | 58 | 0 | 0 | 0 | 0 | 0 | 0 | 0 | 0 | 0 | 0 | 0 |
| **HealtC2** | Male | 41 | 0 | 0 | 0 | 0 | 0 | 0 | 0 | 0 | 0 | 0 | 0 |
| **HealtC3** | Male | 44 | 0 | 0 | 0 | 0 | 0 | 0 | 0 | 0 | 0 | 0 | 0 |
| **HealtC4** | Male | 45 | 0 | 0 | 0 | 0 | 0 | 0 | 0 | 0 | 0 | 0 | 0 |
| **HealtC5** | Male | 60 | 0 | 0 | 0 | 0 | 0 | 0 | 0 | 0 | 0 | 0 | 0 |
| **HealtC6** | Female | 52 | 0 | 0 | 0 | 0 | 0 | 0 | 0 | 0 | 0 | 0 | 0 |
| **HealtC7** | Male | 56 | 0 | 0 | 0 | 0 | 0 | 0 | 0 | 0 | 0 | 0 | 0 |
| **HealtC8** | Male | 49 | 0 | 0 | 0 | 0 | 0 | 0 | 0 | 0 | 0 | 0 | 0 |
| **HealtC9** | Female | 55 | 0 | 0 | 0 | 0 | 0 | 0 | 0 | 0 | 0 | 0 | 0 |
| **HealtC10** | Male | 43 | 0 | 0 | 0 | 0 | 0 | 0 | 0 | 0 | 0 | 0 | 0 |
| **HealtC11** | Female | 57 | 0 | 0 | 0 | 0 | 0 | 0 | 0 | 0 | 0 | 0 | 0 |
| **HealtC12** | Male | 46 | 0 | 0 | 0 | 0 | 0 | 0 | 0 | 0 | 0 | 0 | 0 |

**0 = no symptom; 1 = present symptom.**

**Supplementary Table 2. Key Proteins Involved in Brucellosis**

| **Uniprot Accession** | **Entry Name** | **Protein names** | **Gene Names** | **Organism** | **Length (aa)** |
| --- | --- | --- | --- | --- | --- |
| Q96PD5 | PGRP2_HUMAN | N-acetylmuramoyl-L-alanine amidase (EC 3.5.1.28) (Peptidoglycan recognition protein 2) (Peptidoglycan recognition protein long) (PGRP-L) | PGLYRP2 PGLYRPL PGRPL UNQ3103/PRO10102 | Homo sapiens (Human) | 576 |
|  | extracellular exosome [GO:0070062]; extracellular region [GO:0005576]; membrane [GO:0016020]; N-acetylmuramoyl-L-alanine amidase activity [GO:0008745]; peptidoglycan binding [GO:0042834]; peptidoglycan immune receptor activity [GO:0016019]; zinc ion binding [GO:0008270]; biological process involved in interaction with host [GO:0051701]; defense response to Gram-positive bacterium [GO:0050830]; detection of bacterium [GO:0016045]; innate immune response [GO:0045087]; negative regulation of natural killer cell differentiation involved in immune response [GO:0032827]; negative regulation of type II interferon production [GO:0032689]; peptide amidation [GO:0001519]; peptidoglycan catabolic process [GO:0009253]; regulation of inflammatory response [GO:0050727] | | | | |
| B0AZL7 | B0AZL7_HUMAN | cDNA, FLJ79457, highly similar to Insulin-like growth factor-binding proteincomplex acid labile chain |  | Homo sapiens (Human) | 605 |
|  |  | | | | |
| D9ZGG2 | D9ZGG2_HUMAN | Vitronectin | VTN | Homo sapiens (Human) | 478 |
|  | basement membrane [GO:0005604]; endoplasmic reticulum [GO:0005783]; extracellular space [GO:0005615]; Golgi lumen [GO:0005796]; intracellular membrane-bounded organelle [GO:0043231]; rough endoplasmic reticulum lumen [GO:0048237]; collagen binding [GO:0005518]; extracellular matrix binding [GO:0050840]; heparin binding [GO:0008201]; identical protein binding [GO:0042802]; polysaccharide binding [GO:0030247]; scavenger receptor activity [GO:0005044]; cell-matrix adhesion [GO:0007160]; extracellular matrix organization [GO:0030198]; immune response [GO:0006955]; liver regeneration [GO:0097421]; oligodendrocyte differentiation [GO:0048709]; positive regulation of cell-substrate adhesion [GO:0010811]; protein polymerization [GO:0051258] | | | | |
| A0A384MEF1 | A0A384MEF1_HUMAN | Gelsolin (Actin-depolymerizing factor) (Brevin) |  | Homo sapiens (Human) | 782 |
|  | actin filament binding [GO:0051015]; calcium ion binding [GO:0005509]; actin filament severing [GO:0051014]; actin nucleation [GO:0045010]; barbed-end actin filament capping [GO:0051016]; cell projection organization [GO:0030030] | | | | |
| H0YAC1 | H0YAC1_HUMAN | Plasma kallikrein | KLKB1 | Homo sapiens (Human) | 686 |
|  | extracellular region [GO:0005576]; heme binding [GO:0020037]; iron ion binding [GO:0005506]; monooxygenase activity [GO:0004497]; oxidoreductase activity, acting on paired donors, with incorporation or reduction of molecular oxygen [GO:0016705]; serine-type endopeptidase activity [GO:0004252]; blood coagulation [GO:0007596]; proteolysis [GO:0006508] | | | | |
| P43652 | AFAM_HUMAN | Afamin (Alpha-albumin) (Alpha-Alb) | AFM ALB2 ALBA | Homo sapiens (Human) | 599 |
|  | blood microparticle [GO:0072562]; cytoplasm [GO:0005737]; extracellular exosome [GO:0070062]; extracellular region [GO:0005576]; extracellular space [GO:0005615]; fatty acid binding [GO:0005504]; vitamin E binding [GO:0008431]; zinc ion binding [GO:0008270]; protein stabilization [GO:0050821]; protein transport within extracellular region [GO:0071693]; vitamin transport [GO:0051180] | | | | |
| P10909 | CLUS_HUMAN | Clusterin (Aging-associated gene 4 protein) (Apolipoprotein J) (Apo-J) (Complement cytolysis inhibitor) (CLI) (Complement-associated protein SP-40,40) (Ku70-binding protein 1) (NA1/NA2) (Sulfated glycoprotein 2) (SGP-2) (Testosterone-repressed prostate message 2) (TRPM-2) [Cleaved into: Clusterin beta chain (ApoJalpha) (Complement cytolysis inhibitor a chain); Clusterin alpha chain (ApoJbeta) (Complement cytolysis inhibitor b chain)] | CLU APOJ CLI KUB1 AAG4 | Homo sapiens (Human) | 449 |
|  | apical dendrite [GO:0097440]; blood microparticle [GO:0072562]; cell surface [GO:0009986]; chromaffin granule [GO:0042583]; collagen-containing extracellular matrix [GO:0062023]; cytoplasm [GO:0005737]; cytosol [GO:0005829]; extracellular exosome [GO:0070062]; extracellular region [GO:0005576]; extracellular space [GO:0005615]; Golgi apparatus [GO:0005794]; intracellular membrane-bounded organelle [GO:0043231]; mitochondrial inner membrane [GO:0005743]; mitochondrion [GO:0005739]; neurofibrillary tangle [GO:0097418]; nucleus [GO:0005634]; perinuclear endoplasmic reticulum lumen [GO:0099020]; perinuclear region of cytoplasm [GO:0048471]; platelet alpha granule lumen [GO:0031093]; protein-containing complex [GO:0032991]; spherical high-density lipoprotein particle [GO:0034366]; synapse [GO:0045202]; amyloid-beta binding [GO:0001540]; chaperone binding [GO:0051087]; low-density lipoprotein particle receptor binding [GO:0050750]; misfolded protein binding [GO:0051787]; protein carrier chaperone [GO:0140597]; protein heterodimerization activity [GO:0046982]; protein-containing complex binding [GO:0044877]; signaling receptor binding [GO:0005102]; tau protein binding [GO:0048156]; ubiquitin protein ligase binding [GO:0031625]; unfolded protein binding [GO:0051082]; cell morphogenesis [GO:0000902]; central nervous system myelin maintenance [GO:0032286]; chaperone-mediated protein complex assembly [GO:0051131]; chaperone-mediated protein folding [GO:0061077]; complement activation [GO:0006956]; complement activation, classical pathway [GO:0006958]; immune complex clearance [GO:0002434]; innate immune response [GO:0045087]; intrinsic apoptotic signaling pathway [GO:0097193]; lipid metabolic process [GO:0006629]; microglial cell activation [GO:0001774]; microglial cell proliferation [GO:0061518]; negative regulation of amyloid fibril formation [GO:1905907]; negative regulation of amyloid-beta formation [GO:1902430]; negative regulation of cell death [GO:0060548]; negative regulation of intrinsic apoptotic signaling pathway in response to DNA damage [GO:1902230]; negative regulation of protein-containing complex assembly [GO:0031333]; negative regulation of release of cytochrome c from mitochondria [GO:0090201]; negative regulation of response to endoplasmic reticulum stress [GO:1903573]; positive regulation of amyloid fibril formation [GO:1905908]; positive regulation of amyloid-beta formation [GO:1902004]; positive regulation of apoptotic process [GO:0043065]; positive regulation of gene expression [GO:0010628]; positive regulation of intrinsic apoptotic signaling pathway [GO:2001244]; positive regulation of neurofibrillary tangle assembly [GO:1902998]; positive regulation of neuron death [GO:1901216]; positive regulation of NF-kappaB transcription factor activity [GO:0051092]; positive regulation of nitric oxide biosynthetic process [GO:0045429]; positive regulation of proteasomal ubiquitin-dependent protein catabolic process [GO:0032436]; positive regulation of protein-containing complex assembly [GO:0031334]; positive regulation of receptor-mediated endocytosis [GO:0048260]; positive regulation of tau-protein kinase activity [GO:1902949]; positive regulation of tumor necrosis factor production [GO:0032760]; positive regulation of ubiquitin-dependent protein catabolic process [GO:2000060]; protein import [GO:0017038]; protein stabilization [GO:0050821]; protein targeting to lysosome involved in chaperone-mediated autophagy [GO:0061740]; regulation of amyloid-beta clearance [GO:1900221]; regulation of apoptotic process [GO:0042981]; regulation of cell population proliferation [GO:0042127]; regulation of neuron death [GO:1901214]; regulation of neuronal signal transduction [GO:1902847]; release of cytochrome c from mitochondria [GO:0001836]; response to misfolded protein [GO:0051788]; response to virus [GO:0009615]; reverse cholesterol transport [GO:0043691] | | | | |
| P00734 | THRB_HUMAN | Prothrombin (EC 3.4.21.5) (Coagulation factor II) [Cleaved into: Activation peptide fragment 1; Activation peptide fragment 2; Thrombin light chain; Thrombin heavy chain] | F2 | Homo sapiens (Human) | 622 |
|  | blood microparticle [GO:0072562]; collagen-containing extracellular matrix [GO:0062023]; endoplasmic reticulum lumen [GO:0005788]; extracellular exosome [GO:0070062]; extracellular region [GO:0005576]; extracellular space [GO:0005615]; Golgi lumen [GO:0005796]; plasma membrane [GO:0005886]; serine-type endopeptidase complex [GO:1905370]; calcium ion binding [GO:0005509]; growth factor activity [GO:0008083]; heparin binding [GO:0008201]; lipopolysaccharide binding [GO:0001530]; serine-type endopeptidase activity [GO:0004252]; signaling receptor binding [GO:0005102]; thrombospondin receptor activity [GO:0070053]; acute-phase response [GO:0006953]; antimicrobial humoral immune response mediated by antimicrobial peptide [GO:0061844]; blood coagulation [GO:0007596]; blood coagulation, common pathway [GO:0072377]; cell surface receptor signaling pathway [GO:0007166]; cytolysis by host of symbiont cells [GO:0051838]; fibrinolysis [GO:0042730]; negative regulation of astrocyte differentiation [GO:0048712]; negative regulation of blood coagulation [GO:0030195]; negative regulation of cytokine production involved in inflammatory response [GO:1900016]; negative regulation of fibrinolysis [GO:0051918]; negative regulation of platelet activation [GO:0010544]; negative regulation of proteolysis [GO:0045861]; neutrophil-mediated killing of gram-negative bacterium [GO:0070945]; platelet activation [GO:0030168]; positive regulation of blood coagulation [GO:0030194]; positive regulation of cell growth [GO:0030307]; positive regulation of cell population proliferation [GO:0008284]; positive regulation of collagen biosynthetic process [GO:0032967]; positive regulation of lipid kinase activity [GO:0090218]; positive regulation of phosphatidylinositol 3-kinase signaling [GO:0014068]; positive regulation of phospholipase C-activating G protein-coupled receptor signaling pathway [GO:1900738]; positive regulation of protein localization to nucleus [GO:1900182]; positive regulation of protein phosphorylation [GO:0001934]; positive regulation of reactive oxygen species metabolic process [GO:2000379]; positive regulation of receptor signaling pathway via JAK-STAT [GO:0046427]; positive regulation of release of sequestered calcium ion into cytosol [GO:0051281]; proteolysis [GO:0006508]; regulation of blood coagulation [GO:0030193]; regulation of cell shape [GO:0008360]; regulation of cytosolic calcium ion concentration [GO:0051480]; response to wounding [GO:0009611]; zymogen activation [GO:0031638] | | | | |
| P00747 | PLMN_HUMAN | Plasminogen (EC 3.4.21.7) [Cleaved into: Plasmin heavy chain A; Activation peptide; Angiostatin; Plasmin heavy chain A, short form; Plasmin light chain B] | PLG | Homo sapiens (Human) | 810 |
|  | blood microparticle [GO:0072562]; cell surface [GO:0009986]; collagen-containing extracellular matrix [GO:0062023]; extracellular exosome [GO:0070062]; extracellular region [GO:0005576]; extracellular space [GO:0005615]; extrinsic component of external side of plasma membrane [GO:0031232]; extrinsic component of plasma membrane [GO:0019897]; glutamatergic synapse [GO:0098978]; plasma membrane [GO:0005886]; platelet alpha granule lumen [GO:0031093]; Schaffer collateral - CA1 synapse [GO:0098685]; apolipoprotein binding [GO:0034185]; chaperone binding [GO:0051087]; endopeptidase activity [GO:0004175]; enzyme binding [GO:0019899]; kinase binding [GO:0019900]; protein antigen binding [GO:1990405]; protein domain specific binding [GO:0019904]; serine-type endopeptidase activity [GO:0004252]; serine-type peptidase activity [GO:0008236]; signaling receptor binding [GO:0005102]; biological process involved in interaction with symbiont [GO:0051702]; blood coagulation [GO:0007596]; extracellular matrix disassembly [GO:0022617]; fibrinolysis [GO:0042730]; labyrinthine layer blood vessel development [GO:0060716]; mononuclear cell migration [GO:0071674]; muscle cell cellular homeostasis [GO:0046716]; myoblast differentiation [GO:0045445]; negative regulation of cell population proliferation [GO:0008285]; negative regulation of cell-cell adhesion mediated by cadherin [GO:2000048]; negative regulation of cell-substrate adhesion [GO:0010812]; negative regulation of fibrinolysis [GO:0051918]; positive regulation of blood vessel endothelial cell migration [GO:0043536]; positive regulation of fibrinolysis [GO:0051919]; proteolysis [GO:0006508]; tissue regeneration [GO:0042246]; tissue remodeling [GO:0048771]; trans-synaptic signaling by BDNF, modulating synaptic transmission [GO:0099183]; trophoblast giant cell differentiation [GO:0060707] | | | | |
|  |  | | | | |
